# AMP-activated protein kinase is a key regulator of acute neurovascular permeability

**DOI:** 10.1242/jcs.253179

**Published:** 2021-04-15

**Authors:** Silvia Dragoni, Bruna Caridi, Eleni Karatsai, Thomas Burgoyne, Mosharraf H. Sarker, Patric Turowski

**Affiliations:** 1 Institute of Ophthalmology, University College London, 11-43 Bath Street, London EC1V 9EL, UK; 2 School of Science, Engineering & Design, Teesside University, Stephenson Street, Middlesbrough TS1 3BA, UK

**Keywords:** AMP-activated protein kinase, VEGF-A, Bradykinin, Retina, VE-cadherin, Endothelial nitric oxide synthase

## Abstract

Many neuronal and retinal disorders are associated with pathological hyperpermeability of the microvasculature. We have used explants of rodent retinae to study acute neurovascular permeability, signal transduction and the role of AMP-activated protein kinase (AMPK). Following stimulation with either vascular endothelial growth factor (VEGF-A) or bradykinin (BK), AMPK was rapidly and strongly phosphorylated and acted as a key mediator of permeability downstream of Ca^2+^. Accordingly, AMPK agonists potently induced acute retinal vascular leakage. AMPK activation led to phosphorylation of endothelial nitric oxide synthase (eNOS, also known as NOS3), which in turn increased VE-cadherin (CDH5) phosphorylation on Y685. In parallel, AMPK also mediated phosphorylation of p38 MAP kinases (hereafter p38) and HSP27 (HSPB1), indicating that it regulated paracellular junctions and cellular contractility, both previously associated with endothelial permeability. Endothelial AMPK provided a missing link in neurovascular permeability, connecting Ca^2+^ transients to the activation of eNOS and p38, irrespective of the permeability-inducing factor used. Collectively, we find that, due to its compatibility with small molecule antagonists and agonists, as well as siRNA, the *ex vivo* retina model constitutes a reliable tool to identify and study regulators and mechanisms of acute neurovascular permeability.

## Abstract (precis)

**Summary:** The metabolic sensor protein kinase AMPK is a key regulator of acute retinal vascular permeability by activating endothelial nitric oxide synthase and p38 MAPK.

## INTRODUCTION

Leakage within the vascular system can be the cause or a significant co-morbidity of a variety of pathologies and is the consequence of endothelial hyperpermeability, which leads to extravasation of fluids and proteins, resulting in interstitial oedema (Nagy et al., 2008; Claesson-Welsh et al., 2021). In the nervous system, where the vasculature is uniquely impermeable and is referred to as the blood–brain barrier (BBB), vascular leakage accompanies stroke, multiple sclerosis, Parkinson’s disease as well as various forms of dementia (Turowski, 2017; Sweeney et al., 2019). Neurovascular leakage also affects the blood–retinal barrier (BRB), where it is a hallmark feature of diabetic retinopathy and neovascular age-related macular degeneration (Klaassen et al., 2013). Leakage during retinopathies is driven by permeability-inducing factors (PIFs), most prominently by the angiogenic growth factor VEGF-A, and VEGF-A antagonists are successfully used to reduce oedema and abnormal vessel growth, and to restore neuronal dysfunction (Brown et al., 2013). Meta-analysis of the clinical use of anti-VEGFs in diabetic macular oedema suggests that PIFs other than VEGF-A play an important role in the pathogenesis of retinal leakage disease (Ford et al., 2013), including angiopoietin-2 (Campochiaro and Peters, 2016), lysophosphatidylcholine (Canning et al., 2016) and bradykinin (BK; Kita et al., 2015).

PIFs induce both acute and chronic vascular leakage. For instance, exposure of the vascular endothelium to vascular endothelial growth factor (VEGF-A) leads to acute permeability that usually lasts less than 30 min. If not resolved, persistent leakage ensues, which chronically impairs vascular integrity (Nagy et al., 2008; Bates, 2010; Claesson-Welsh et al., 2021). Different PIFs bind to distinct endothelial cell (EC) surface receptors, but ultimately all permeability responses involve paracellular junction modulation or formation of transport vesicles (Nagy et al., 2008; Turowski, 2017; Claesson-Welsh et al., 2021), suggesting that ECs regulate hyperpermeability through a core molecular machinery and common downstream signalling. Indeed, Ca^2+^ transients, phosphorylation of the p38 MAP kinases (hereafter p38) and enhanced actin contractility are associated with all vascular permeability, as is activation of eNOS (also known as NOS3; Wu et al., 1999; Hudson et al., 2014; Weidert et al., 2014; di Lorenzo et al., 2013; Ninchoji et al., 2020 preprint). Adherens- and tight-junction modulation is associated with paracellular permeability and the phosphorylation of VE-cadherin (VE-cad, also known as CDH5) is associated with acute permeability in the periphery and the retina (Orsenigo et al., 2012; Smith et al., 2020). In the retina, the phosphorylation of occludin on S490 and its subsequent internalisation also plays an important role, at least in a more chronic setting (Murakami et al., 2012). Identifying core signalling, on which all leakage responses depend, is desirable for therapeutic development as it would allow treatment without prior knowledge of a specific known (or unknown) extracellular PIF.

In the brain and the retina, entirely different signalling is induced by VEGF-A in ECs when it is added luminally (from the blood side) or abluminally (from the tissue side), with leakage-inducing signalling entirely restricted to abluminal stimulation (Hudson et al., 2014). For instance, leakage-associated p38 activation is triggered by abluminal (basal) VEGF-A stimulation, whereas activation of the phosphoinositide 3-kinase (PI3K)–AKT pathway, which does not mediate permeability, is only seen following luminal (apical) stimulation. Thus, signalling specific to leakage can be inferred by comparing cellular stimulation following luminal and abluminal addition of VEGF-A. Conversely, BK efficiently induces permeability from the basal as well as apical side of cerebral or retinal ECs.

AMP-activated protein kinase (AMPK) is a phylogenetically conserved energy sensor that regulates energy homeostasis by coordinating metabolic pathways and thus balancing energy requirement with nutrient supply (Hardie, 2018). Previous studies suggest that AMPK acts as a protector of BBB integrity, for instance by preventing lipopolysaccharide (LPS)-enhanced NAD(P)H oxidase expression in ECs and the consequent barrier dysfunction and enhanced permeability (Zhao et al., 2014; Ange et al., 2020). Moreover, AMPK mediates upregulation of BBB functions induced *in vitro* by metformin, a drug used for the treatment of diabetes (Takata et al., 2013). Nevertheless, in the retina AMPK activation can lead to the breakdown of the outer, non-vascular BRB, which is constituted by the retinal pigment epithelium (Villarroel et al., 2011); however, if and how AMPK contributes to permeability induction by agonists such as VEGF-A or BK in neural microvessels is unknown.

To identify and validate core components mediating acute permeability in neurovascular ECs, we adopted an *ex vivo* retinal preparation, originally described for rats (Warboys et al., 2009). Development of this method allowed measurement of real-time changes of permeability and signalling in intact BRB vessels from rat and mouse. Importantly, this model system was compatible with precise pharmacokinetic agonist studies, parallel immunohistochemical staining and manipulation using siRNA. Our workflow can be used to identify core regulators of central nervous system (CNS) endothelial hyperpermeability and was validated by identifying AMPK as a novel, key regulator linking VEGF-A or BK-induced Ca^2+^ transients to eNOS activation and VE-cad phosphorylation.

## RESULTS

### VEGF-A and BK-induced permeability and junctional changes in brain microvascular ECs

**Fig. 1.**
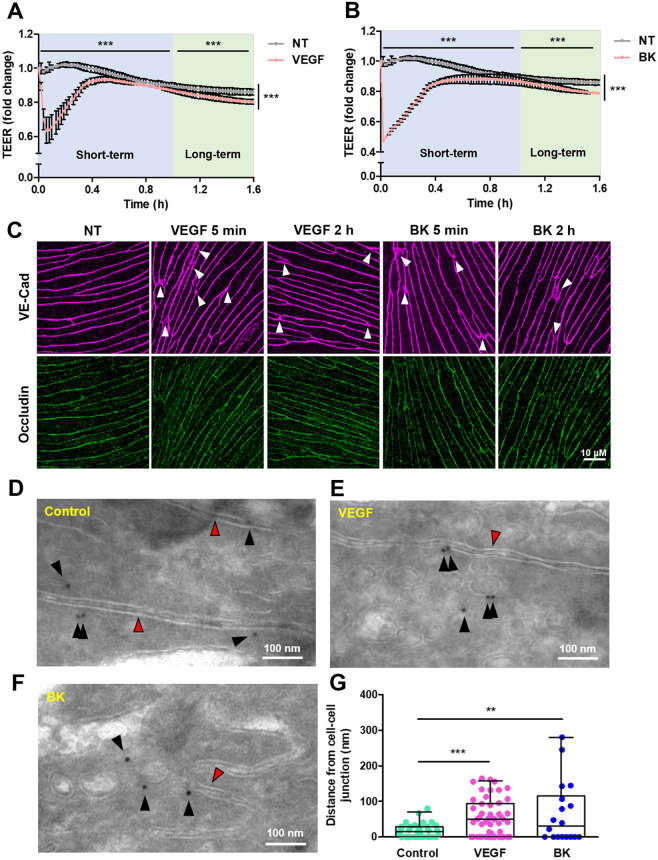
**VEGF-A- and BK-induced permeability in cultured brain microvascular ECs.** (A,B) Primary rat brain microvascular ECs were grown on permeable Transwell inserts to confluence and until they reached full electrical barrier (500–800 Ω cm^2^). VEGF-A (A) or BK (B) were added at time 0. Mean±s.d. normalised resistance changes are shown for treated and non-treated (NT) ECs (*n*=3). Significant changes were detected in the short- and long-term, as well as the overall responses. (C) Changes in the distribution of VE-cad and occludin in response to basal (corresponding to abluminal) stimulation with VEGF-A or BK were analysed by confocal microscopy in post-confluent primary rat brain microvascular ECs. White arrowheads indicate broadening of the VE-cad staining compared to that in the NT control. Scale bar: 10 μm. (D–F) Cryo-immuno EM of VE-cad distribution in control (D) and VEGF-A-stimulated (E) or BK-stimulated (F) human hCMEC/D3 cells. Shown are inter-endothelial junction areas with the two adjacent membranes (red arrowheads). Black arrowheads point to gold labelled VE-cad, which in control cells was found predominantly associated with abutting plasma membranes (within 20 nm; i.e. the distance expected by the primary and the secondary bridging antibody). Scale bars: 100 nm. (G) Box and whisker plot of distances between VE-cad gold particles and cell–cell junctions, as determined from three independent preparations as shown in D–F. The interquartile range and median are indicated by the box and line, respectively, with whiskers indicating the range. Individual points are also shown. **P<0.01; ***P<0.001 (two-way ANOVA with post-hoc Bonferroni's multiple comparison test in A,B; one-way ANOVA with post-hoc Dunnett's tests in G).

Treatment of primary rat brain microvascular ECs with VEGF-A or BK significantly reduced transendothelial electrical resistance (TEER), indicating that paracellular permeability was induced (Fig. 1A,B). TEER dropped immediately and reached a minimum within less than 5 min after addition of either VEGF-A or BK before reverting to control levels within 1 h. Thereafter, another significant, but more modest reduction in TEER was observed, indicative of a more chronic change in cell monolayer permeability. In order to correlate TEER changes with paracellular junction breakdown, the distribution of occludin and VE-cad was analysed in VEGF-A- and BK-stimulated primary brain microvascular ECs (Fig. 1C). As judged by confocal microscopy, occludin expression and distribution remained unchanged for up to 2 h of VEGF-A or BK stimulation. VE-cad levels also remained unchanged, but a broadening of the staining was observed within 5 min of the addition of the PIF, in particular at and around tricellular junctions. In agreement, cryo-immunogold electron microscopy (cryo-immuno EM) of hCMEC/D3 cells revealed a significant relocation of VE-cad from the junctions to the cell interior by an average distance of 55 and 66 nm following a 5 min stimulation with VEGF-A and BK, respectively (Fig. 1D–G). These results showed that single addition of either VEGF-A or BK induced acute and chronic permeability, and that the acute response was accompanied by VE-cad redistribution away from the junctions.

### Validation of a modified *ex vivo* retinal model

**Fig. 2.**
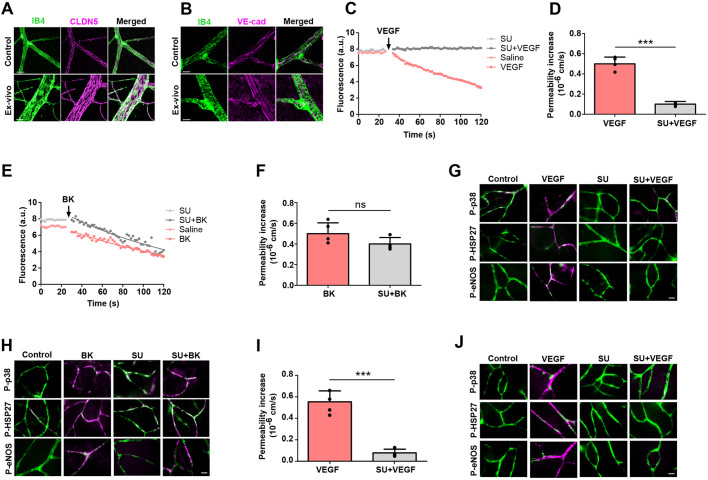
**Validation of the *ex vivo* retina model in rats.** (A,B) Control retinae were from animals directly perfused and fixed with 4% PFA. *Ex vivo* retinae were isolated, flat mounted and left submerged with Krebs solution for 1 h before PFA fixation. Whole mounts were stained with isolectin B4 (IB4), and anti-claudin-5 (CLDN5) and anti-VE-cad, as indicated. (C–F) Sulforhodamine B fluorescence intensities were recorded in single retinal capillaries. At the times indicated (arrows in C,E), 50 ng/ml VEGF-A (C,D) or 10 μM BK (E,F) were applied on top of the retina (abluminal side). Optionally, retinae were pre-incubated with the VEGFR2-selective antagonist SU-1498 (10 μM; SU) for 15 min prior to recording. Saline treatment was used as a negative control. Lines in C,E were fitted as described in the Materials and Methods. Independent data points and mean±s.d. permeability changes are shown in D and F (a.u., arbitrary units). (G,H) *Ex vivo* retinal explants were preincubated without or with 10 μM SU for 15 min as indicated, then stimulated with VEGF-A (G) or BK (H) for 2 min, then fixed using 4% PFA before staining with IB4 (green) and for phospho-p38 (pT180/Y182; P-p38), phospho-HSP27 (pS82; P-HSP27) or phospho-eNOS (pS1177; P-eNOS) (magenta). (I) Microvessel permeability changes were recorded in mouse retinae as described in C,D. (J) Mouse retinal explants were stimulated and stained as described in G using phospho-specific antibodies to p38, HSP27 and eNOS. ns, not significant; ***P<0.001 (paired, two-tailed *t*-test). Scale bars: 10 µm.

In order to study the acute phase of VEGF-A- and BK-induced permeability in intact retinal microvessels in real time, we adopted an *ex vivo* retinal model (Warboys et al., 2009). Permeability measurements used rat retinal explants, in which the vasculature was stabilised with a cardioplegic solution. To assess whether the *ex vivo* preparation and perfusions led to alterations of the retinal vasculature and to determine stability of the preparation, we compared directly perfused fixed retinae with others perfused with cardioplegic solution and left under superfusion with Krebs solution for 1 h before fixation. Subsequent whole-mount staining for the tight junction protein claudin-5 (CLDN5) and the adherens junction protein VE-cad revealed characteristic strands of continuous paracellular staining (Fig. 2A,B) in both preparations. Importantly, the staining pattern was indistinguishable between the two different preparations, indicating that the perfusion did not cause significant disturbances of endothelial junctions. Permeability measurements were carried out by monitoring sulforhodamine B loss from individual microvessels (Fig. S1A,B). Baseline permeability to sulforhodamine B was very low and on average 0.2±0.16×10^−6^ cm/s (mean±s.d.). Taken together, these data showed that morphological and barrier properties of the retinal microvasculature were well preserved in these preparations.

Stimulation of the *ex vivo* retina with VEGF-A or BK induced an immediate, marked loss of sulforhodamine B from the microvessel lumen, which was similar for both stimuli and amounted to ∼3-fold increase in microvessel permeability (Fig. 2C–F). Pre-incubation of the *ex vivo* retina with VEGFR2 (also known as KDR) inhibitor SU-1498 for 15 min prevented VEGF-A- but not BK-induced permeability, confirming the role of VEGFR2 in VEGF-A-induced permeability and indicating that BK acted through a different receptor. Whole-mount retinal staining showed that VEGF-A- and BK-induced permeability coincided with the phosphorylation of p38 on T180 and/or Y182, its downstream effector HSP27 (also known as HSPB1; phosphorylated on S82), as well as eNOS (on S1177) (Fig. 2G,H). Phosphorylation of all three downstream effectors in response to VEGF-A but not BK was abolished following pre-incubation with SU-1498.

This experimental model was also used for mouse retinae. Baseline permeability in mouse preparations was 0.15±0.1×10^−6^ cm/s (mean±s.d.). VEGF-A stimulation increased permeability to 0.65±0.2×10^−6^ cm/s, and this was again sensitive to SU-1498 (Fig. 2I). Furthermore, we observed SU-1498-sensitive phosphorylation of p38, HSP27 and eNOS in mouse retinal microvessels within 2 min of VEGF-A stimulation (Fig. 2J). To assess the compatibility of the *ex vivo* retina with knockdown technology, mouse eyes were injected intravitreously with siRNA against CLDN5. Western blot analysis of retinal lysates, harvested 72 h after the injection, showed that CLDN5 expression was significantly reduced by 65% (Fig. S1C,D), and this was corroborated by whole-mount staining of the retina (Fig. S1E). Microvessel permeability following knockdown of CLDN5 increased ∼3-fold (to 0.41±0.03 cm/s; Fig. S1F,G). Taken together, these results demonstrated that the *ex vivo* retinal preparation was a reliable model to measure retinal paracellular microvessel permeability in rats and mice.

### VEGF-A and BK induce AMPK phosphorylation

**Fig. 3.**
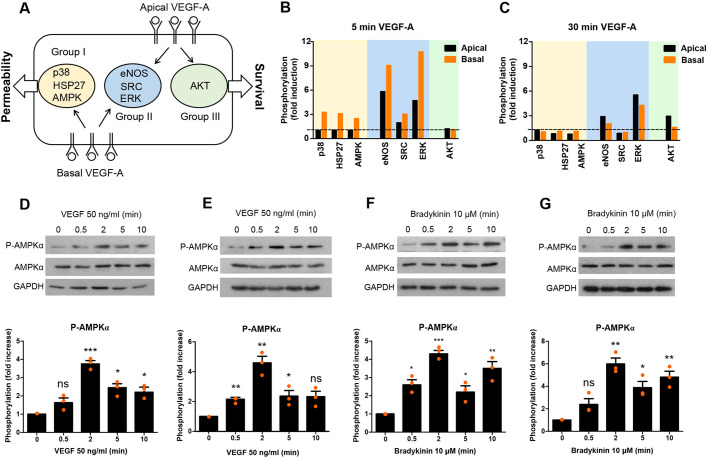
**VEGF-A-** **and** **BK-induced AMPK phosphorylation.** (A–C) Primary brain microvascular ECs were stimulated with VEGF-A from the apical or basal side for 5 min, triggering apically specific (group I), basally specific (group III) or mixed (group II) responses (see Results and Hudson et al., 2014 for more details). (A) Schematic of VEGF-A response groups. (B,C) Cells were lysed after the indicated VEGF-A treatments, and phosphorylation of the indicated molecules in the lysates was assessed by phosphoantibody array analysis. (D–G) Brain microvascular ECs (D,F) or *ex vivo* rat retinae (E,G) were stimulated with 50 ng/ml VEGF-A (D,E) or 10 μM BK (F,G) for the indicated length of time, and AMPKα phosphorylation (pT172; P-AMPKα) was analysed. Representative results and quantification of AMPKα phosphorylation from three independent experiments are shown. Quantitative data are presented as normalised mean±s.d. *P<0.05; **P<0.01; ***P<0.001; ns, not significant (one-way ANOVA with post-hoc Dunnett's tests).

In order to find new regulators of permeability, primary rat brain microvascular ECs were stimulated for 5 or 30 min with VEGF-A (50 ng/ml) from either the apical (non-permeability inducing) or basal (permeability-inducing) side (Fig. 3A), and cell lysates were analysed using a phosphoprotein antibody array. In response to VEGF-A stimulation, many signalling components were phosphorylated, as exemplified by phosphorylation of p38, HSP27, AMPK, eNOS, SRC, ERK (collectively referring to ERK1 and ERK2, also known as MAPK3 and MAPK1, respectively) and AKT (referring to AKT1, 2 and 3) (Fig. 3B,C).

Differentially phosphorylated proteins were categorised into three groups: group I, which were phosphorylated only after basal stimulation with VEGF-A (including p38, HSP27 and AMPK); group II, which were phosphorylated regardless of the side of the stimulation (such as eNOS, SRC and ERK); and group III, which were phosphorylated only when VEGF-A was applied apically (such as AKT). Phosphorylation of proteins exclusively in response to basally applied VEGF-A suggested they played a role in hyperpermeability (group I). Among these, AMPK stood out, because its role in acute endothelial hyperpermeability has not yet been studied in detail. Additionally, AMPK was among the 10 proteins for which phosphorylation increased the most in response to basally applied VEGF-A, when analysed by phosphopeptide mass spectrometry (S.D. and P.T., unpublished results). A previous study has also demonstrated that AMPK links Ca^2+^ transients to VE-cad phosphorylation in response to ICAM-1 engagement in brain microvascular ECs (Martinelli et al., 2009).

AMPK phosphorylation in response to VEGF-A was confirmed by western blot analysis. Stimulation of primary rat brain ECs from the basal side led to rapid, transient phosphorylation of the catalytic subunit AMPKα on T172, which peaked after ∼2 min (Fig. 3D). A very similar activation pattern was observed in the intact rat retina when VEGF-A was applied directly to the top of the isolated retina (corresponding to the basal side of the endothelium; Fig. 3E). BK induced similar phosphorylation of AMPKα T172, both in the cultured primary brain microvascular ECs and intact retina (Fig. 3F,G). It was notable that AMPK phosphorylation was maximal after ∼2 min in response to both VEGF-A and BK; however, BK clearly induced more sustained phosphorylation.

### AMPK mediates VEGF-A- and BK-induced permeability

**Fig. 4.**
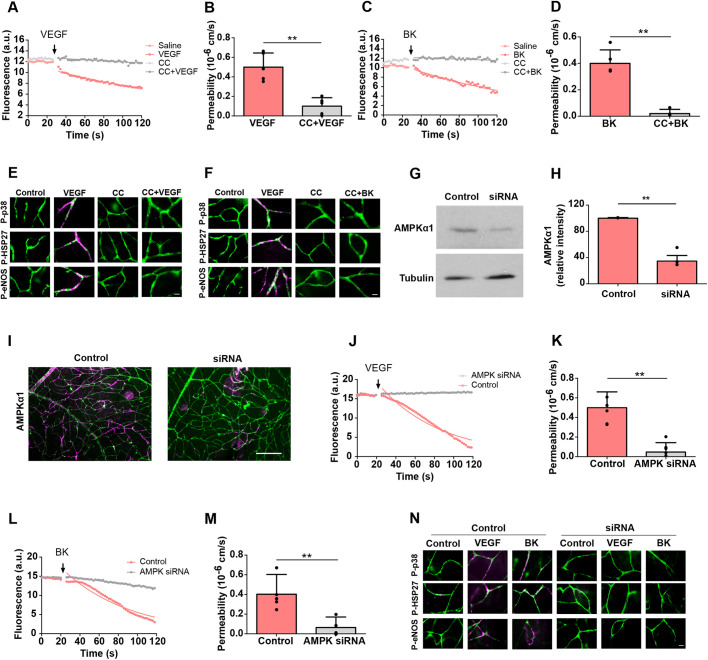
**AMPK-mediated VEGF-A- or BK-induced permeability.** (A–F) Rat *ex vivo* retinae were pre-incubated with or without compound C (CC, 10 μM) for 15 min, then 50 ng/ml VEGF-A (A,B) or 10 μM BK (C,D) was applied to the top of the retina at the indicated time (arrows in A,C), and changes in permeability were recorded. Saline treatment was used as a control. Alternatively (E,F), retinae were immunostained usin IB4 (green) and anti-phospho-p38 (P-p38), anti-phospho-HSP27 (P-HSP27) or anti-phospho-eNOS (P-eNOS) antibodies (magenta), as detailed in Fig. 2. (G–N) AMPKα1-specific siRNA or scrambled control was injected into the vitreous of mouse eyes. (G) After 72 h, retinae were isolated, lysed and subjected to immunoblotting using AMPKα1- and tubulin-specific antibodies. (H) Densitometric quantification of three independent immunoblots as shown in G. Control and knockdown retinae were also prepared for *ex vivo* permeability measurements and stimulated using VEGF-A (50 ng/ml) (J,K) or BK (10 μM) (L,M). Alternatively, retinae were fixed and immunostained using IB4 (green) and anti-AMPKα1 (I), anti-phospho-p38, anti-phospho-HSP27 or anti-phospho-eNOS antibodies (magenta) (N). Note that neither PIF induced any permeability in the AMPKα1 knocked-down retina. Representative results and quantifications from independent experiments are shown. Data in B,D,H,K,M are presented as mean±s.d. Lines in A,C,J,L were fitted as described in the Materials and Methods. a.u., arbitrary units. **P<0.01 (paired, two-tailed *t*-test). Scale bars: 10 μm.

In order to specify the role of AMPK during VEGF-A- and BK-induced vascular permeability, AMPK activity was neutralised in the *ex vivo* retina. Pre-incubation of the *ex vivo* retina with compound C, a widely used AMPK antagonist, significantly decreased VEGF-A- and BK-induced permeability by 80% and 93%, respectively (Fig. 4A–D). Moreover, whole-mount staining showed that pre-treatment with compound C prevented the VEGF-A- and BK-induced activation of p38, HSP27 and eNOS (Fig. 4E,F).

Brain and retinal endothelial cells express AMPKα1 (also known as PRKAA1), but little to no AMPKα2 (PRKAA2) (Vanlandewijck et al., 2018; Lipski et al., 2020). To assess neutralisation of AMPK more specifically, AMPKα1 was knocked down by intraocular injection of siRNA 72 h prior to preparing the retinae for further experimentation. Knocked-down retinae contained ∼65% less AMPKα1 (Fig. 4G,H), with a substantial loss in the vasculature as seen by immunohistochemistry (Fig. 4I). AMPKα1 siRNA also reduced retinal AMPKα overall by ∼70% (Fig. S1H,I), indicating that AMPKα1 was the predominant isoform in the retina. In AMPKα1 knocked-down retinae, neither VEGF-A nor BK stimulation led to significant permeability (Fig. 4J–M), or the phosphorylation of p38, HSP27 or eNOS (Fig. 4N).

**Fig. 5.**
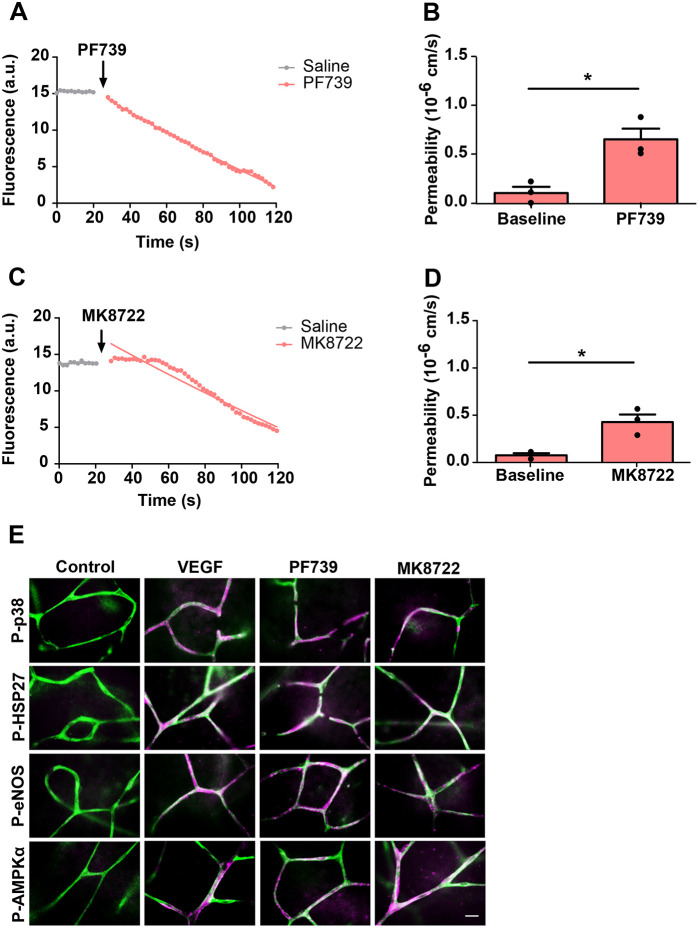
**Stimulation of AMPK induces permeability in the *ex vivo* retina.** (A–D) *Ex vivo* preparations were stimulated with the two different AMPK activators (A,B) PF739 (10 μM) and (C,D) MK8722 (10 μM). Saline treatment was used as a control. Both agonists induced strong and immediate permeability in the *ex vivo* retinal microvessels. Lines in A,C were fitted as described in the Materials and Methods. Data in B,D are mean±s.d. permeability changes recorded from three retinae. (E) *Ex vivo* retinae were stimulated as in A–D, or by addition of 50 ng/ml VEGF-A, and after 2 min fixed using 4% PFA and then immunostained using IB4 (green) and anti-phospho-p38 (P-p38), anti-phospho-HSP27 (P-HSP27), anti-phospho-eNOS (P-eNOS) or anti-phospho-AMPKα (P-AMPKα) antibodies (magenta), as detailed in Fig. 2. *P<0.05 (paired, two-tailed *t*-test). Scale bar: 10 µm.

Retinal endothelial cells express AMPKβ1 (also known as PRKAB1) and AMPKβ2 (PRKAB2) (Lipski et al., 2020), and are thus susceptible to AMPK activation by a wide variety of molecules binding to its allosteric drug and metabolite (ADaM) site (Hardie, 2017). Stimulation of *ex vivo* retinae with the AMPKβ1 and AMPKβ2-specific activators PF739 and MK8722 (Fig. 5A–D), or the reportedly less potent, AMPKβ1-specific activator A769662 (Fig. S2A,B) led to instantaneous loss of intravascular rhodamine, with average increases in permeability of ∼6.5-, 5- and 3-fold, respectively. All three compounds also induced substantial phosphorylation of AMPK, p38, HSP27 and eNOS in retinal microvessels within 2 min of stimulation (Fig. 5E; Fig. S2E). In addition, 5-aminoimidazole-4-carboxamide riboside (AICAR), a classically used compound that, once inside cells, is phosphorylated to generate an AMP analogue with some specificity for AMPK (Kim et al., 2016), led to a similar increase in permeability and phosphorylation of AMPK, p38, HSP27 and eNOS (Fig. S2C–E). Taken together these results confirmed the central role of AMPK in VEGF-A- and BK-induced acute retinal leakage and showed that AMPK acted upstream of p38 and eNOS.

### VEGF-A- and BK-induced permeability requires Ca^2+^, CAMKK, p38 and eNOS

**Fig. 6.**
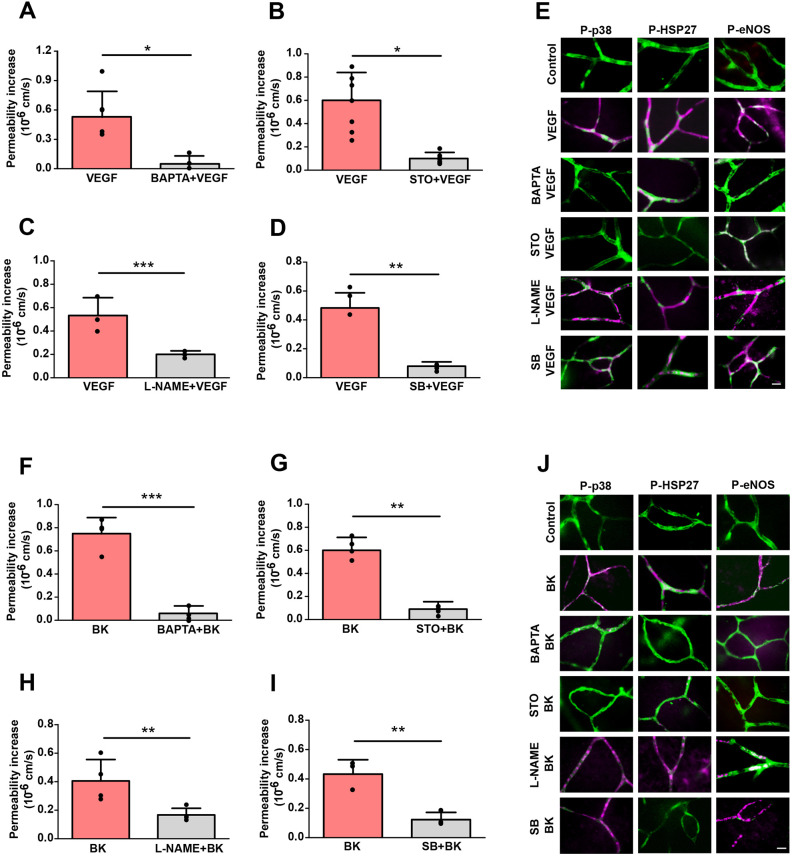
**VEGF-A- and BK-induced permeability requires Ca^2+^, CaMKK, p38 and eNOS.** (A–E) *Ex vivo* retinae were pre-incubated with (A) 20 µM BAPTA (Ca^2+^ chelator), (B) 10 µM STO-609 (STO; CaMKK inhibitor), (C) 10 µM L-NAME (NOS inhibitor) or (D) 10 µM SB202190 (SB; p38 inhibitor) for 15 min. Then VEGF-A (50 ng/ml) was applied to the top of the retina (abluminal side) and changes in microvessel permeability were recorded, as described in Fig. 2. (E) Alternatively, treated and control retinae, as indicated, were fixed after 2 min using 4% PFA and then immunostained with IB4 (green) and for phosphorylation of p38 (P-p38), HSP27 (P-HSP27) and eNOS (P-eNOS) (magenta). (F–J) As in panels A–E, except that *ex vivo* retinae were stimulated with BK (10 μM) instead of VEGF-A. Representative results and quantification (independent data points and means±s.d.) are shown. *P<0.05; **P<0.01; ***P<0.001 (paired, two-tailed *t*-test). Scale bars: 10 μm.

Next, we aimed to place AMPK within established PIF signalling cascades. Ca^2+^ is critical for the activation of both p38 and eNOS (Takeda et al., 2004; Martinelli et al., 2009; da Silva et al., 2009). To test its role in VEGF-A-induced vascular leakage, the *ex vivo* retina was incubated with BAPTA, a Ca^2+^ chelant, prior to VEGF-A administration. BAPTA treatment significantly reduced VEGF-A-induced permeability by 94%, and also prevented the phosphorylation of p38, HSP27 and eNOS (Fig. 6A,E), suggesting that Ca^2+^ acted upstream to these molecules. CAMKK (herein referring to all isoforms) is able to phosphorylate and activate AMPK in a Ca^2+^-dependent manner (Martinelli et al., 2009; Hurley et al., 2005). Treatment of retinae with the CAMKK inhibitor STO-609 significantly reduced VEGF-A-induced permeability by 86% and also prevented the activation of p38, HSP27 and eNOS (Fig. 6B,E). The role of eNOS in permeability was re-confirmed by pre-incubation of the *ex vivo* retinae with L-NAME, a NOS inhibitor, which reduced VEGF-A-induced permeability by 87% but did not have any effect on p38 or HSP27 phosphorylation (Fig. 6C,E), indicating that eNOS was not upstream of p38. Finally, the *ex vivo* retina was pre-incubated with the p38 inhibitor SB203580, which significantly reduced the VEGF-A-induced permeability by 80%, as well as activation of HSP27 but not of eNOS (Fig. 6D,E).

Similar results were obtained for BK-induced stimulation of retinae. Incubation with either BAPTA or STO-609 nearly completely abolished BK-induced permeability, together with the activation of p38, HSP27 and eNOS (Fig. 6F,G,J). Pre-treatment of the *ex vivo* retina with L-NAME reduced BK-induced permeability by 67% but did not affect p38 or HSP27 phosphorylation (Fig. 6H,J). Finally, treatment with SB203580 prevented BK-induced permeability, as well as phosphorylation of HSP27 but not eNOS (Fig. 6I,J).

**Fig. 7.**
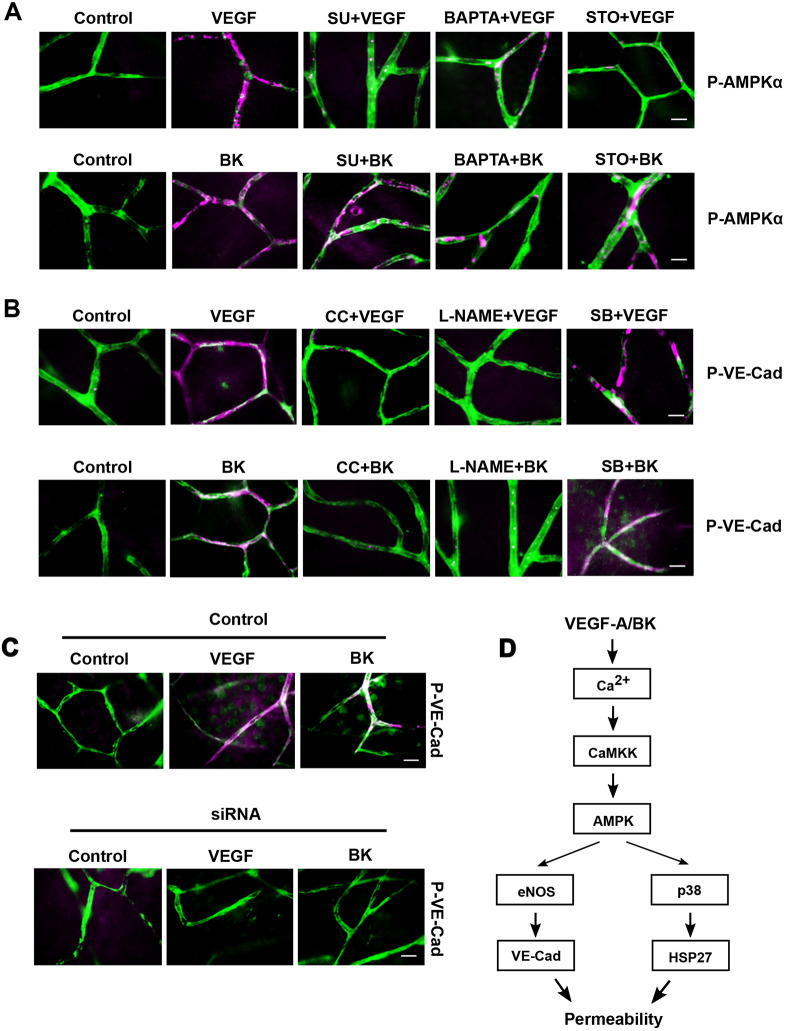
**VEGF-A- and BK-induced VE-cadherin phosphorylation.** (A) *Ex vivo* retinae were pre-incubated with SU-1498 (10 µM; SU), BAPTA (20 µM) or STO-609 (10 µM; STO) and treated with VEGF-A or BK for 2 min. Retinae were then fixed with 4% PFA and immunostained for phospho-AMPKα (magenta; P-AMPKα) and blood vessels (using IB4, green). (B) *Ex vivo* retinae were pre-incubated with compound C (10 µM; CC), L-NAME (10 µM) or SB202190 (10 µM; SB), and treated with VEGF-A or BK for 2 min, then immunostained for phosphorylated VE-cad (using anti-pY685-VEC; P-VE-Cad; magenta) and blood vessels (using IB4, green). (C) AMPKα1-specific siRNA or scrambled control were injected into the vitreous of mouse eyes. After 72 h, retinae were treated with VEGF-A or BK for 2 min and immunostained for phosphorylated VE-cad (using anti-pY685-VEC; magenta) and blood vessels (using IB4, green). (D) Proposed signalling networks in the *ex vivo* retinal microvasculature downstream of VEGF-A and BK. Scale bars: 10 µm.

Importantly, VEGF-A- or BK-induced phosphorylation of AMPKα was completely abolished by treatment with BAPTA or STO-609. VEGF-A- but not BK-induced AMPKα phosphorylation was also abolished by SU-1498 (Fig. 7A). These results indicated that in the retina, VEGF-A and BK induced Ca^2+^ transients and consequent activation of AMPK via CAMKK. At this point, signalling diverged into either activation of p38 or eNOS, which both contributed to permeability (Fig. 7D).

### VEGF-A and BK stimulate VE-cadherin phosphorylation

Lastly, we wanted to analyse VE-cad internalisation in the VEGF-A and BK stimulated *ex vivo* retina; however, systematic correlation of cryo-immuno EM to leaky microvessels proved impractical. We therefore used phosphorylation of VE-cad on Y685 as a surrogate marker for retinal microvessel permeability (Smith et al., 2020), in particular because such VE-cad phosphorylation is also a prerequisite for internalisation after BK treatment (Orsenigo et al., 2012). VEGF-A or BK stimulation of *ex vivo* retinae induced tyrosine phosphorylation of VE-cad on Y685 in intact microvessels (Fig. 7B). This phosphorylation was completely abolished following pre-incubation with compound C or L-NAME, but not SB203580. The specific involvement of AMPK in VEGF-A- or BK-induced VE-cad phosphorylation was further confirmed in eyes injected intraocularly with AMPKα1-targetting siRNA (Fig. 7C). Taken together, these results showed that that VE-cad modulation was downstream of AMPK–eNOS but not AMPK–p38 (Fig. 7D). Indeed, direct activation of *ex vivo* retinae with the nitric oxide (NO) donor DEANO also induced VE-cad phosphorylation (Fig. S3A).

## DISCUSSION

Measuring retinal microvascular permeability has been mainly restricted to Miles-type assays using either fluorescent tracers or Evans Blue (binding to plasma albumin) (Radu and Chernoff, 2013; Canning et al., 2016). Chronic leakage can also be visualised in real time by fluorescein angiography (Couturier et al., 2019). However, collectively these methods, which measure the amount of extravasated tracer, do not only reflect the degree of leakage but are also strongly influenced by dye concentration in the vasculature and dye clearance from the tissue (Nagy et al., 2008; Turowski, 2017). Further disadvantages of these methods are that compound concentrations and timing cannot be controlled accurately. Thus, they are inadequate to measure acute permeability and associated signalling accurately and in a controlled manner.

The *ex vivo* retinal platform described herein addressed most of these issues. It constituted a significant advance to EC cultures, since it used a complete and intact neurovascular unit. The functionality of the retinal vasculature was preserved, with both VE-cad and claudin-5 distribution indistinguishable from that *in vivo*. Similar preparations of the brain and the retina also display full cellular functionality of, for example, pericytes (Mishra et al., 2014). Importantly, permeability to sulforhodamine B was very low and within the range of that of other small non-ionic molecules at the intact BBB *in vivo* (Shi et al., 2014) and notably ∼10-fold lower than in preparations of pial microvessels (Easton et al., 1997), indicating that this model was highly suitable for permeability measurement of an intact neurovascular unit. Vascular barrier properties in these retinal explants was, as expected, dependent on tight junction integrity. The reported speed at which compounds such as VEGF-A induce permeability (Bates, 2010) was fully recapitulated. Leakage measurements were then combined with whole-tissue staining and analyses of the phosphorylation status of key mediators of permeability using phospho-specific antibodies to gain mechanistic insight. Furthermore, the preparations could be interrogated using small molecule antagonists and agonists at defined concentrations and times, allowing the identification of key downstream regulators common to both VEGF-A and BK stimulation. Even more specific neutralisation of key proteins was achieved through prior intraocular injection of siRNA. Conceivably, this model is compatible for use with genetically modified mice and disease models, further broadening its applicability. The *ex vivo* platform could also be used to measure Ca^2+^ transients or localised production of reactive oxygen or nitrogen species in response to vasoactive compounds such as VEGF-A or BK.

Measurements in retinae were made *ex vivo*, in the absence of blood flow. Blood flow and associated shear stress may influence EC biology, such as cell–cell adhesion and inflammatory dysfunction (Orsenigo et al., 2012; Chistiakov et al., 2017; Ho et al., 2019) and their absence in our preparation must be taken into account when evaluating results. It should, however, also be noted that permeability regulation by shear stress appears to be remembered in ECs *in vitro* for at least 24 h (Walsh et al., 2011).

For the purpose of developing and validating the *ex vivo* retinal platform we have focused on PIFs that induce permeability when added to the abluminal (tissue) side of the endothelium, such as VEGF-A and BK (Hudson et al., 2014), as these are readily applied on top of the retinal explants. Other PIFs that act only from the luminal side, such as lysophosphatidic acid (Hudson et al., 2014) or lysophosphatidylcholine (Canning et al., 2016), could also potentially be investigated in this system; however, this would require the use of a manifold injection system (Liu et al., 2000), which allows switching between injection of sulforhodamine B with or without permeability factor into radial vessels.

VEGF-A and BK induced acute leakage in retinal microvessels, which was associated with and dependent on Ca^2+^, the MAPK p38 and eNOS, in agreement with published data (Wu et al., 1999; Hudson et al., 2014; di Lorenzo et al., 2013). We also identified AMPK, specifically its catalytic subunit isoform AMPKα1, as a novel key mediator of both VEGF-A- and BK-induced permeability, indicating that it is a core regulator of acute vascular permeability. AMPK is primarily known to regulate the energy requirements of the cell, but has also been implicated in other seemingly unrelated cellular processes such as migration, cell growth and apoptosis (Hardie, 2011). This protein kinase has been studied previously in relation to its protective role at the BBB, in particular when challenged by LPS (Takata et al., 2013; Zhao et al., 2014; Ange et al., 2020), whereas in the retinal pigment epithelium it has been shown to be responsible for permeability induced by IL-1β (Villarroel et al., 2011). However, all these studies address chronic changes and do not focus on the role of AMPK for acute permeability. Whereas canonical activation of AMPK is dependent on cellular AMP:ATP concentration ratio and phosphorylation on T172 by LKB1 (also known as STK11), we found that, in the regulation of endothelial permeability, AMPK was activated downstream of Ca^2+^ and CAMKK. This activation pathway has previously been described as non-canonical (Carling et al., 2008) and is also operational when CNS ECs facilitate the transmigration of lymphocytes (Martinelli et al., 2009). Notably, VEGF-A has also been reported to induce NO production via a pathway requiring Ca^2+^ and AMPK (Reihill et al., 2007). Indeed, eNOS phosphorylation on S1177 can be mediated by AKT (Fulton et al., 1999) or AMPK (Martinelli et al., 2009; Youn et al., 2009). However, the PI3K–AKT pathway is not relevant for VEGF-A-induced permeability in neurovascular ECs (Hudson et al., 2014). We confirmed the phosphorylation of eNOS on S1177 downstream of AMPK both during VEGF-A and BK permeability induction. Notably, the Ca^2+^–AMPK–eNOS pathway resulted in the phosphorylation of VE-cad on Y685, identified previously as key for vascular permeability in the periphery (Orsenigo et al., 2012) and the retina (Smith et al., 2020), thus providing a direct link between AMPK and paracellular junction regulation. Although in our system NO-induced VE-cad phosphorylation was sensitive to the soluble guanylyl cyclase inhibitor ODQ (Fig. S3A), suggesting a connecting role for cGMP and protein kinase G, adherens junction complexes also appear to be direct targets of NO during permeability induction, namely through S-nitrosylation of  b-catenin (CTNNB1) and its subsequent dissociation from VE-cad (Thibeault et al., 2010). Further research is required to determine how protein kinase G and direct S-nitrosylation processes are functionally linked to the tyrosine phosphorylation and internalisation of VE-cad. For retinal permeability, occludin phosphorylation and internalisation also play an important role; however, judging from the published timecourses, it is likely to be a later event, not captured by our experiments, and thus either secondary to VE-cad phosphorylation or, with its dependency on PKCβ (PRKCB) activation, outside of the signalling network we have investigated here (Murakami et al., 2012).

In response to VEGF-A and BK, AMPK also regulated the phosphorylation of the MAPK p38 and its substrate HSP27, both previously implicated in actin rearrangement during endothelial barrier disruption (Sawada et al., 2015; Kása et al., 2015; Hirano et al., 2004). p38 is a bona fide regulator of VEGF-A responses (Corre et al., 2017), and its activation downstream of CDC42 and p21-activated protein kinases (PAKs) and subsequent modulation of the actin cytoskeleton occurs during VEGF-A-induced endothelial migration downstream of VEGFR2 phosphorylation on Y1214 (Lamalice et al., 2004). The PAK1 isoform regulates VEGF-induced permeability in cultured mouse endothelial cells, possibly through direct phosphorylation of VE-cad (Gavard and Gutkind, 2006). However, FRAX597, a potent group I PAK inhibitor (Han et al., 2020), did not reduce permeability induction or p38 phosphorylation of the VEGF-A- or PF739-stimulated *ex vivo* retina (Fig. S3B-E). This points to potentially important molecular differences between permeability regulation in neural and non-neural endothelium. In the absence of evidence for a role of PAK proteins, we favour an alternative model of direct activation of p38 by AMPK via TAB1, a pathway described in apoptotic lymphocytes and the ischemic heart (Li et al., 2005; Lanna et al., 2014). By switching between two different p38 activation modes (Ca^2+^–AMPK–TAB1 versus CDC42–PAK) ECs could adapt cytoskeletal regulation to the specific requirement of EC migration or permeability. However, this model requires thorough future analysis with specific focus on all endothelially expressed PAK and TAB isoforms.

The *ex vivo* retina proved to be a reliable model and demonstrated its usefulness in identifying key regulators of acute permeability. Although AMPK clearly emerged as such a key regulator, it is unlikely to be exploitable as a target for anti-leakage treatments: its central role in regulating cellular energy demands throughout the body hints at many potential side effects. Activation of AMPK is currently investigated as a therapeutic option to treat cancer, metabolic syndrome and diabetes (Hardie, 2017; Kim et al., 2016). However, in light of the strong induction of permeability we observed in response to at least two AMPK agonists, we propose that these avenues should be explored cautiously, because at least acute microvascular leakage may accompany such treatment modalities. Nevertheless, our data collectively indicate that the *ex vivo* retina platform can play an important part in elucidating mechanisms and signalling of neurovascular leakage.

## MATERIALS AND METHODS

### Materials

Recombinant rat VEGF-A (165) was purchased from R&D Systems (Abingdon, United Kingdom). Bradykinin, sulforhodamine B, Evans Blue, SU-1498, SB203580, compound C, STO-609, L-N^G^-nitroarginine methyl ester (L-NAME), BAPTA-AM, 5-aminoimidazole-4-carboxamide ribonucleotide (AICAR), A769662, diethylammonium (Z)-1-(N,N-diethylamino)diazen-1-ium-1,2-diolate (DEANO) and 1H-[1,2,4]-oxadiazolo[4,3-a]quinoxalin-1-one (ODQ) were purchased from MERCK (Dorset, United Kingdom). PF739 was purchased from AOBIOUS (Gloucester, USA). MK8722 was purchased from Cambridge Biosciences (Cambridge, UK). FRAX597 was purchased from Stratech Scientific Ltd (Ely, UK). Polyclonal antibodies specific for p38, HSP27, AMPKα, eNOS and their phosphorylated forms (p38 Thr180/Tyr182, HSP27 Ser82, AMPK Thr172 and eNOS Ser1177) were from Cell Signaling Technology (Beverly, MA). Recombinant Anti-AMPK alpha 1 antibody was from Abcam (Cambridge, UK). Polyclonal antibodies against phosphorylated VE-cad (p-Y658-VEC and p-Y685-VEC) were a gift from Dr Fabrizio Orsenigo and Professor Elisabetta Dejana (FIRC Institute of Molecular Oncology, Milan and Uppsala University). Anti-occludin and claudin-5 antibodies were from ThermoFisher Scientific. Affinity purified rabbit anti-VE-cad antibodies have been described previously (Martins et al., 2013). Fluorescein-labelled isolectin B4 was from Vector Laboratories (FL-1201). Further details of antibodies are provided in Table S1.

### Animals

Wistar female rats (7–10 weeks old) and C75BL/6J mice (7–12 weeks old) were purchased from Charles River Laboratories. All animal procedures were performed in accordance with Animal Welfare Ethical Review Body (AWERB) and Association for Research in Vision and Ophthalmology (ARVO) Statement for the Use of Animals in Ophthalmic and Vision Research guidelines and under a UK Home Office licence.

### Brain microvascular EC isolation and culture

Microvessels were isolated from rat cortical grey matter by collagenase dispase digestion and BSA and Percoll density gradient centrifugation (Hudson et al., 2014). Purified vessels were seeded onto collagen IV- and fibronectin-coated Costar Transwells (Fisher Scientific) at high density (vessels from six rat brains per 40 cm^2^). Cells were grown in EGM2-MV (Lonza), with 5 mg/ml puromycin from the second day for 3 days, for 2–3 weeks until their TEER plateaued at values above 200 Ω cm^2^.

The human brain microvascular EC line hCMEC/D3 was also grown in EGM2-MV as previously described (Weksler et al., 2005).

### Transendothelial electrical resistance

Changes in the TEER were determined by time-resolved impedance spectroscopy of primary cerebral rat brain microvascular ECs grown on 12 mm Transwells, using a CellZscope (Nanoanalytics). Before the addition of VEGF-A and BK, TEER values were 500–800 Ω cm^2^.

### Immunocytochemistry

Primary cerebral rat brain microvascular ECs were fixed with methanol (−20°C). Staining was performed as previously described using antibodies against occludin (1:100; OC-3F10, Invitrogen; Turowski et al., 2004) or VE-cad (1:100; Martins et al., 2013).

### Immunogold electron microscopy

Cryo-immuno EM was performed as previously described (Hudson et al., 2014). Briefly, hCMEC/D3 cells were fixed in 4% paraformaldehyde (PFA) and 0.1% glutaraldehyde. Sections were stained using antibodies against the extracellular domain of VE-cad (1:30; Serotech). Image J (NIH) was used to process images and measure the distances between the gold particles and the interendothelial junctions.

### Retinal explant preparation

Retinal explants were prepared essentially as described before (Warboys et al., 2009). A Wistar female rat or C75BL/6J mouse was killed by overdose of CO_2_. The common carotid artery was carefully exposed and cannulated with a glass microcannula. The head vasculature was then flushed, first with heparinised saline (300 U/l heparin in 0.9% NaCl; mouse, 5 ml; rat, 20 ml), then with stabilising solution (10 mM Mg^2+^, 110 mM NaCl, 8 mM KCl, 10 mM HEPES and 1 mM CaCl_2_, pH 7.0, with 10 µM isoproterenol added before use; mouse, 5 ml; rat, 20 ml), also referred to as cardioplegic solution containing isoproterenol, and finally with the same solution supplemented with 5 g/l Evans Blue dye in 10% albumin (mouse, 5 ml; rat, 20 ml) for subsequent visualisation of the vasculature. Next, an eye was surgically removed and the retina isolated, together with the attached sclera. The retina was flattened onto a transparent silicone medium (SYLGARD 184 SILICONE ELASTOMER KIT by Dow Corning), kept in position by a metal ring, and the resulting well was sealed with grease. Throughout the procedure the retina was continuously superfused with Krebs solution (124 mM sodium chloride, 5 mM potassium chloride, 2 mM MgSO_4_, 0.125 mM NaH_2_PO_4_, 22 mM NaHCO_3_ and 2 mM CaCl_2_, pH 7.4, with 5 mM glucose and 0.1% BSA added before use).

### Permeability measurements

Retinal explants were mounted for visualisation and further experimentation on an upright Zeiss Axiophot fluorescence microscope. A radial vessel of a superfused retinal explant was cannulated at ∼150 μm from the optic nerve using a microinjection needle (tip diameter 1–5 µm, sharpened to a bevel of <30°) and the entire retinal vasculature injected with sulforhodamine B (1 mg/ml in Krebs solution). Illumination was switched to fluorescence, and the vessels were visualised under a TRITC filter using an Olympus 40× water immersion objective. For permeability measurements, a microvessel was chosen at least 200 µm away from the cannulated radial vessel. Fluorescent content of the vessel was recorded continuously by time-lapse imaging (1 frame/2 s) on a Hamamatsu CCD camera for at least 2 min. A baseline was recorded for ∼30 s, before VEGF-A or BK (in Krebs solution) was added on the top of the retina. Time-lapse series were analysed using ImageJ. Time-dependent fluorescence intensity data of the chosen vessel was derived from a square region of interest (∼18×18 pixels; Fig. S1A,B). Fluorescence in the immediate vicinity of the microvessel was measured and subtracted from the vessel fluorescence measurements. Pixel intensity measurements were charted against time, and permeability values were computed by fitting data to the exponential equation *C_t_*=*C*_0_×e^−*kt*^, where *k*=4*P*/*d* and *d* is the diameter of the vessel (Hudson et al., 2014). The difference in permeability between pre-treatment and post-treatment resulted in the absolute permeability change associated with the treatment regimen.

### Immunohistochemistry

After dissection, retinae from rat or mouse were fixed with 4% PFA at room temperature for 1 h. After 30 min of blocking (3% Triton X-100, 1% Tween and 0.5% BSA in 2× phosphate-buffered saline), retinae were incubated with primary antibodies (1:100) against isolectin B4 (IB-4), claudin-5, and phosphorylated forms of p38, HSP27, eNOS, AMPKα, and VE-cadherin at 4°C overnight. Retinae were washed and incubated with matching Alexa Fluor-conjugated secondary antibodies at room temperature for 2 h. Finally, retinae were washed and mounted using Mowiol 4-88 mounting medium (Sigma). More details can be found in Hudson et al. (2014).

### siRNA-mediated knockdown of claudin-5 and AMPKα1

Specific siRNA targeting claudin-5 was a gift from Dr Matthew Campbell (Trinity College Dublin). siRNA targeting the α1 subunit of AMPK (PRKAA1) was purchased from Dharmacon (Chicago, IL). Mice were anaesthetised by intraperitoneal injection of 100 μl of 6% Narketan (ketamine) and 10% Dormitor (medetomidine) in sterile water. A 2 µl volume of siRNA (1 ng/ml in sterile phosphate-buffered saline) was injected intravitreously in the right eye under a stereomicroscope, using a Hamilton syringe with a 3° Hamilton RN needle (Esslab). As a control, 2 µl of scrambled siRNA was injected into the left eye. To inject, an initial puncture was made to the superior nasal sclera, at the level of the pars plana. Then, the tip of the needle was further introduced through the puncture hole with a 45° angle into the vitreous body. Retinae were isolated 72 h after the injection.

### Phosphoantibody array

Mature monolayers of primary, unpassaged brain microvascular ECs grown on 24 mm Costar Transwells were stimulated with VEGF-A (50 ng/ml) from the apical or basal side for 5 or 30 min. Cells from two Transwells were combined and subjected to screening using a Human Phospho-Kinase Array Proteome Profiler Array (R&D Systems; ARY003B) exactly according to the manufacturer's instructions. Arrays were exposed for varying amounts of time to capture signals in the linear range and quantified using densitometric scanning and ImageJ (NIH). Signals were normalised using array-internal controls. Results were expressed as fold differences between apical versus basal signals.

### Western blotting

Cell lysates were prepared as previously described (Hudson et al., 2014). Proteins were separated by SDS–PAGE and transferred to nitrocellulose by semidry electrotransfer. Membranes were blocked overnight and then incubated with the appropriate primary antibody diluted at 1:2000. Membranes were washed three times with Tris-buffered saline containing 0.1% Tween-20 before 1 h incubation with an HRP-conjugated anti-mouse or anti-rabbit IgG secondary antibody (GE Healthcare) at a dilution of 1:10,000 or 1:5000, respectively. Membranes were developed using the ECL reagents (Roche) and exposed to X-ray film. Protein bands were evaluated by densitometric quantification, normalised against the amount of total protein, and either GADPH or tubulin. A selection of raw blots is shown in Fig. S4.

### Statistics

For each experiment, a protocol was written following careful consideration of the total number of animals or samples and group sizes required to realistically achieve the objectives based on both relevant publications and previous experience of the model used. Accordingly, we performed power (0.8) calculations (http://powerandsamplesize.com) to determine the anticipated number of animals or samples needed. These were discussed with an in-house statistician. Generally, the statistical model used was ANOVA (pairwise, two-sided equality). Effect size and s.d. were guided by our previous extensive accumulated data. We aimed to achieve a significance level of 5% and a power of 80% to minimise the number of animals per experimental group. All raw data was used for analyses. If the morphological integrity of tissue explants appeared compromised, no further experiments were performed with such samples. All attempts of replication were successful, and the data shown represents mean or aggregate data from all replications. Explants from mice or rat were randomly assigned to an experimental protocol. Each experiment on a single tissue explant consisted of a treatment and an appropriate control. Image analyses were conducted by different experimenters, who did not know the identity of the treatment when categorising responses.

TEER measurements of three independent cell monolayers were combined and expressed as mean±s.d. Significant differences were determined by two-way ANOVA with replication, with significance levels set at 0.05, followed by post-hoc Bonferroni's multiple comparison test.

Distances of VE-cad immunogold complexes were measured in electron micrographs from three independent preparations, averaged and expressed as mean±s.d. Significant differences were determined by one-way ANOVA with significance levels set at 0.05, followed by post-hoc Dunnett's tests.

Densitometric quantification of three independent immunoblots were determined by changes in protein or phosphoprotein content normalised to tubulin or GADPH total protein loading controls, with values expressed as fold increase. Data were presented as mean±s.d. Statistics were performed using one-way ANOVA with significance levels set at 0.05, followed by post-hoc Dunnett's tests.

Permeability measurements from at least three different *ex vivo* retinal preparations were combined and expressed as mean±s.d. Significant differences were determined via paired, two-tailed *t*-test between the control and each inhibitor.

Significance levels were set to *P<0.05; **, 0.001<*P*<0.01, ***P≤0.001.

## Supplementary Material

Supplementary information

Reviewer comments
