# Supplementary material for: AMP-activated protein kinase is a key regulator of acute neurovascular permeability
Source: J Cell Sci. 2021 Apr 15;134(7):jcs253179. doi: 10.1242/jcs.253179 (PMC8077405; doi:10.1242/jcs.253179)
Supplement: Supplementary information [file joces-134-253179-s1.pdf]

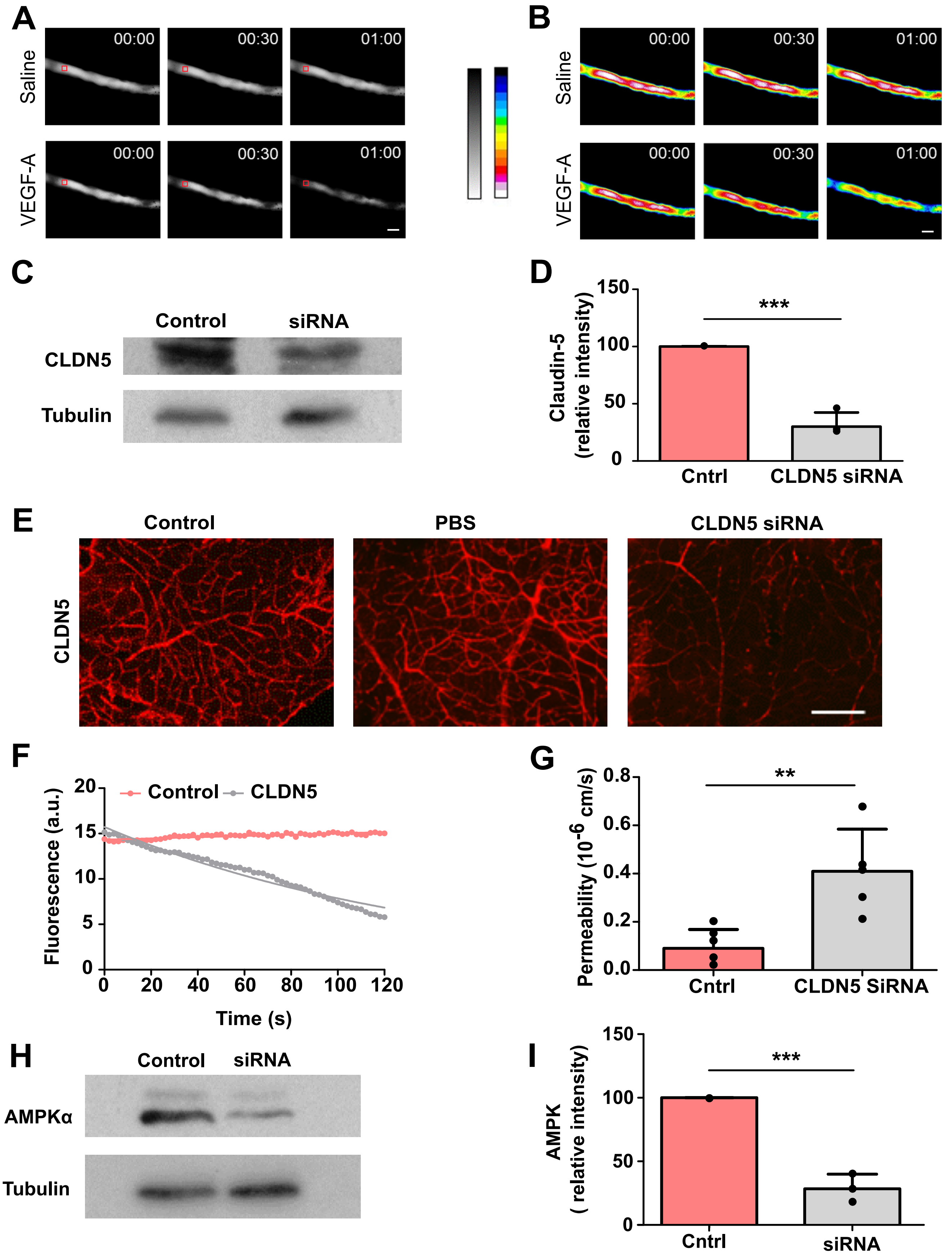

Figure S1. Properties of the ex vivo retina model. (A-B) Selected frames, pseudocoloured in (B), of time-resolved recordings of a Sulforhodamine-B-filled rat capillary before and after the addition of 50 ng/ml VEGF-A, illustrating the rapid loss of fluorophore from the lumen of the vessel. The red box exemplifies a typical r.o.i used for intensity measurement. Scale bars, 10 µm. **(C-G)** CLDN5 siRNA was injected into mouse eyes. 72 h later CLDN5 levels were analysed by immunoblots of retinal lysates (C-D) or by wholemount immunochemical staining (E). Scale bar, 100 µm. Alternatively, permeability of 4 kDa Rhodamine was measured in ex vivo retinae from CLDN5 siRNA or control injected eyes (F), with quantifications (independent data points, means ± SD) shown in (G). **(H, I)** AMPKα1 specific siRNA or scrambled control was injected into the vitreous of mouse eyes. After 72 h retinae were isolated, lysed and subjected to immunoblotting as indicated using anti-pan-AMPKα-specific and -tubulin antibodies (H). Shown in (I) is the densitometric quantification of 3 independent experiments as shown in (H). **$p < 0.01$, ***$p < 0.001$.

Journal of Cell Science • Supplementary information

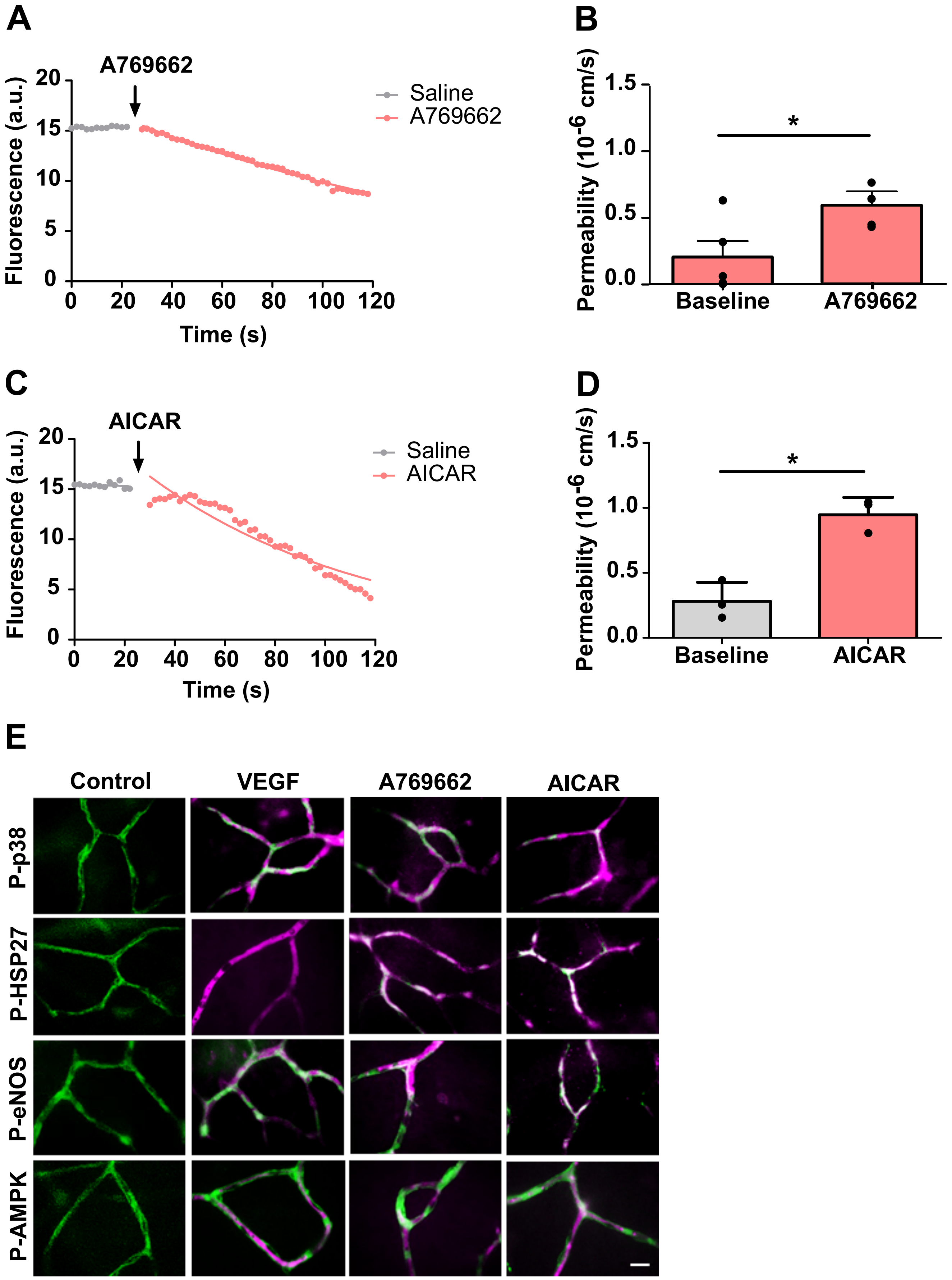

Figure S2. Additional AMPK stimulation experiments in the ex-vivo retina. (A-D) Ex vivo preparations were stimulated with the AMPK activators A769662 (10 µM, A-B) and AICAR (10 µM, C-D). Both agonists induced strong and immediate permeability in the ex vivo retinal microvessels. Mean (± SD) permeability changes recorded from three retinae are shown in (B, D). **(E)** Ex vivo retinae were stimulated as in (A-D) and after 2 min fixed using 4% PFA and then immunostained using IB4 (green) and or anti-phospho-p38, -HSP27 or -eNOS antibodies (magenta) as detailed in Figure 2. *p < 0.05. Scale bars, 10 µm.

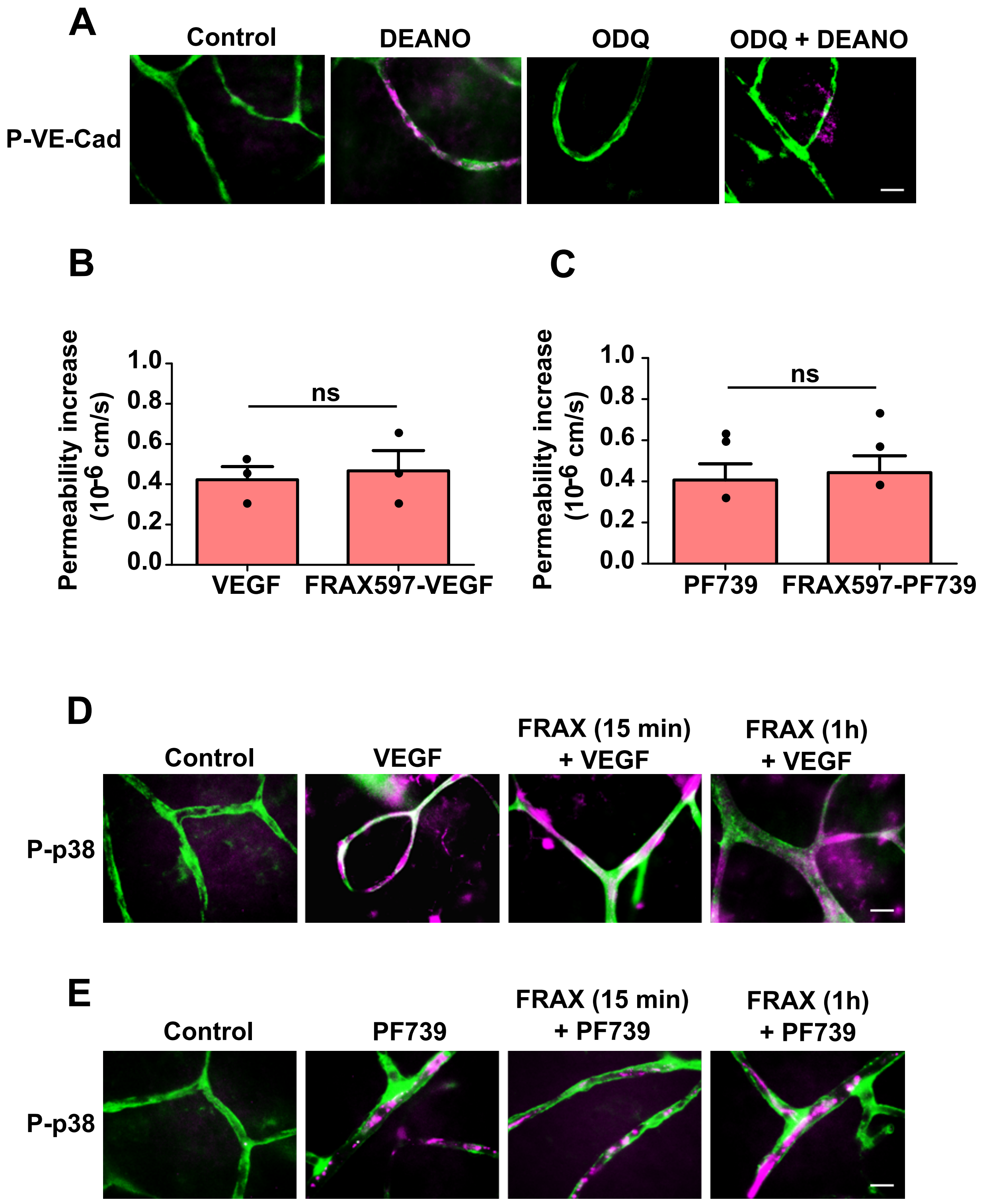

Figure S3. Sensitivity of AMPK-dependent permeability signalling to inhibitors of soluble guanylyl cyclase and PAK. (A) Ex vivo preparations were pre-incubated with or without ODQ (10 µM) for 15 min and then stimulated with the NO donor DEANO (10 µM). After 5 min, the ex-vivo retinae were fixed using 4% PFA and immunostained using IB4 (green) and for phospho-VE-Cad (magenta). **(B, C)** Ex vivo retinae were preincubated with or without FRAX597 (10 µM) for 15 min and stimulated with the VEGF-A (10 ng/ml, B) or the AMPK activator PF739 (10 µM, C). Shown are mean (± SD) permeability changes recorded from three retinae (ns, non signficant). **(D, E)** Ex vivo retinae were stimulated with or without FRAX597 (10 µM) for 15 min or 1h, stimulated with VEGF-A (10 ng/ml, D) or PF739 (10 µM, E) for 5 min, fixed using 4% PFA and then immunostained using IB4 (green) and for phospho-p38 (magenta). Scale bars, 10 µm. Note that FRAX597 was used at ca. 1000 x of the reported IC50 for PAK1-3. Other, similar ATP-binding site inhibitors used in this study, e.g. SB202190, were highly effective in the ex vivo retina at significantly lower relative inhibitory concentrations (200 x IC50), indicating that absence of FRAX597 effects were unlikely due to insufficient PAK targeting.

Journal of Cell Science • Supplementary information

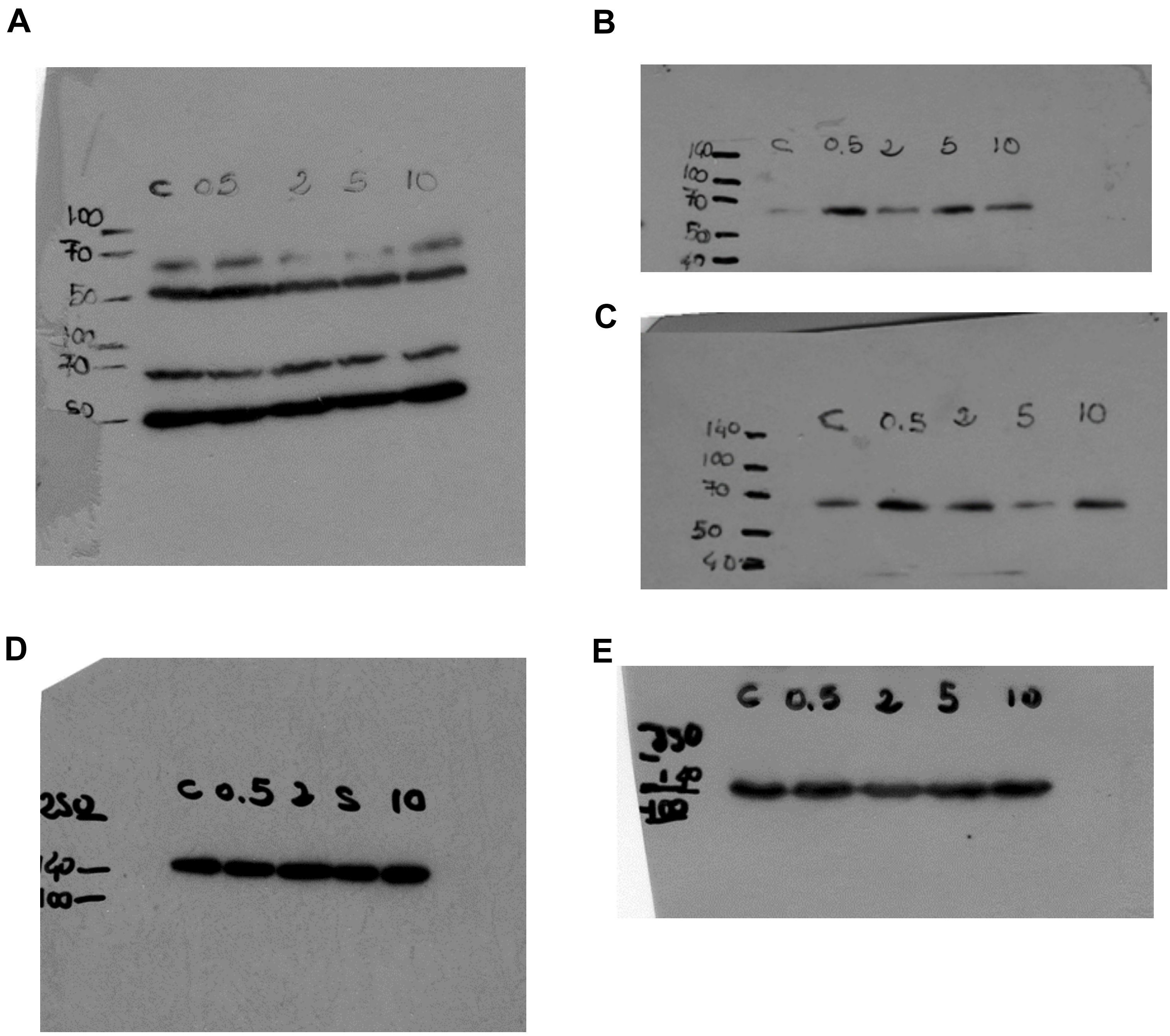

Figure S4. Samples of original western blots. To optimize our samples and save animals, we cut the membrane based on the molecular weight and we obtained stripes that we incubated with different antibodies. AMPK (60 kDa) and Tubulin (55 kDa) have a similar molecular weight. We tried to blot them together but the bands were too close (A) and to obtain nicer figures we opted for GAPDH (144 kDa) (D, E). Samples of P-AMPK (60 kDa) are shown in (B, C). Lanes 1 to 5 for all membranes: Control – 0.5 min – 2 min – 5 min – 10 min VEGF-A (10ng/ml).

Journal of Cell Science • Supplementary information

Table S1

Antibodies used for immunofluorescence (IF), immunohistochemistry (IHC), western blots (WB) or cryo-immuno EM (EM)

**Antibody**	**Species**	**Dilution**	**Company (catalogue #)/reference**
VE-Cad	Rabbit	1:100 (IF)	Martins et al., 2013
Occludin	Rabbit	1:100 (IF)	ThermoFisher Scientific (33-1500)
Claudin 5	Mouse	1:100 (IHC) 1:1000 (WB)	ThermoFisher Scientific (35-2500)
Phospho-p38 (Thr180/Tyr182)	Rabbit	1:100 (IHC)	Cell Signalling (9211)
Anti-Phospho-HSP27 (Ser82)	Rabbit	1:100 (IHC)	Cell Signalling (2401)
Phospho-eNOS (Ser1177)	Rabbit	1:100 (IHC)	Cell Signalling (9570)
Phospho-AMPKα (Thr172)	Rabbit	1:100 (IHC) 1:1000 (WB)	Cell Signalling (2535)
AMPKα	Rabbit	1:1000 (WB)	Cell Signalling (2532)
AMPKα1	Rabbit	1:1000 (WB)	Abcam (ab32047)
GADPH	Mouse	1:1000 (WB)	Cell signalling (5174)
αTubulin	Mouse	1:10000 (WB)	Merck (T9026)
Phospho-Y658-VEC	Rabbit	1:100 (IHC)	Orsenigo et al., 2012
VE-Cad (extracellular domain)	Rabbit	1:30 (EM)	Serotech (AHP628Z)

Journal of Cell Science • Supplementary information
