# Supplementary material for: AMP-activated protein kinase is a key regulator of acute neurovascular permeability
Source: J Cell Sci. 2021 Apr 15;134(7):jcs253179. doi: 10.1242/jcs.253179 (PMC8077405; doi:10.1242/jcs.253179)
Supplement: Reviewer comments [file joces-134-253179_review_history.pdf]

Journal of Cell Science

AMP-activated protein kinase is a key regulator of acute neurovascular permeability

Silvia Dragoni, Bruna Caridi, Eleni Karatsai, Thomas Burgoyne, Mosharraf H. Sarker and Patric Turowski
DOI: 10.1242/jcs.253179

Editor: John Heath

Review timeline

Original submission: 17 August 2020
Editorial decision: 30 October 2020
First revision received: 11 February 2021
Accepted: 2 March 2021

Original submission

First decision letter

MS ID#: JOCES/2020/253179

MS TITLE: AMP-activated protein kinase is a key regulator of acute neurovascular permeability

AUTHORS: Silvia Dragoni, Bruna Caridi, Eleni Karatsai, Thomas Burgoyne, Mosharraf H Sarker, and Patric Turowski
ARTICLE TYPE: Research Article

We have now reached a decision on the above manuscript.

To see the reviewers' reports and a copy of this decision letter, please go to: https://submit-jcs.biologists.org and click on the 'Manuscripts with Decisions' queue in the Author Area. (Corresponding author only has access to reviews.)

As you will see, the reviewers raise a number of substantial criticisms that prevent me from accepting the paper at this stage. They suggest, however, that a revised version might prove acceptable, if you can address their concerns. If you think that you can deal satisfactorily with the criticisms on revision, I would be pleased to see a revised manuscript. We would then return it to the reviewers.

We are aware that you may be experiencing disruption to the normal running of your lab that makes experimental revisions challenging. If it would be helpful, we encourage you to contact us to discuss your revision in greater detail. Please send us a point-by-point response indicating where you are able to address concerns raised (either experimentally or by changes to the text) and where you will not be able to do so within the normal timeframe of a revision. We will then provide further guidance. Please also note that we are happy to extend revision timeframes as necessary.

Please ensure that you clearly highlight all changes made in the revised manuscript. Please avoid using 'Tracked changes' in Word files as these are lost in PDF conversion.

I should be grateful if you would also provide a point-by-point response detailing how you have dealt with the points raised by the reviewers in the 'Response to Reviewers' box. Please attend to

© 2021. Published by The Company of Biologists under the terms of the Creative Commons Attribution License (https://creativecommons.org/licenses/by/4.0/).

all of the reviewers' comments. If you do not agree with any of their criticisms or suggestions please explain clearly why this is so.

Reviewer 1

Advance summary and potential significance to field

Vascular permeability is of high clinical significance. Early signs for diabetic or hypertensive retinopathy are areas with increased vascular permeability. The secondary endpoint of the anti-VEGF-A therapy for wet age-related macular degeneration, macular thickness, is based on the assumption of VEGF-A driven vascular permeability. However, although the concept of vascular permeability is proven in the clinic, its physiological base is not understood.
The manuscript by Dragoni and colleagues presents work that identifies a central VEGF-A- and bradykinin-driven pathway that results in increased permeability in the endothelial cell-to-cell junctions. Using direct measurements of vascular permeability the paper presents interesting data from well performed experiments that fully support the final conclusions.

Comments for the author

Minor comments:
1. The permeability increase by VEGF-A or BK: The authors present a couple of data of permeability increase by VEGF-A from different rows of experiments that focus on the different levels of signal transduction. In almost all panels the mean permeability increase was in between 0.55 and 0.6 that fits nicely into the variance. However, in figures 6C and 6D VEGF-A leads to permeability increases by 1.6 and 0.3 respectively. Are these differences caused by the experimental conditions that did not become clear from the figure caption? Or is there a mistake in the y-axis labeling? The same applies for the effects of BK that were between 0.3 and 0.5. However in the figures 6H and 6F the BK effects were at 0.8 and 1.2 respectively. May be in general that figure legends could be improved for readability.
2. Statistics: The statistics chapter in the Methods section state that there were used four independent experiments but the figure legends state that these were three independent experiments. Furthermore, the dots in the box plot indicate here and there higher “n-numbers” than three. Can it be that from each of the three independent experiments not only one but several blood vessels were analyzed?
3. Westernblots: As the authors show only very small parts of a complete westernblot it would be useful to add complete blots (from each AMPKalpha tubulin, GAPDH one blot) to the supplemental material to demonstrate that the antibodies work well and specifically (only one band at the right molecular mass marker).

Reviewer 2

Advance summary and potential significance to field

The manuscript by Dragoni et al uses an ex vivo model of retina explants to investigate the role of AMPK in neurovascular permeability. The authors confirm previous studies showing that VEGF and bradykinin activate AMPK via CAMKK2 signalling, and report that this leads to increased vascular leakage. Downstream of AMPK, increased phosphorylation of eNOS, p38, HSP27 and VE-cadherin was observed. Using mainly pharmacological approaches, the authors show that inhibition of CAMKK2, eNOS or p38 reduces VEGF and bradykinin induced permeability. Based on their findings, the authors suggest a model whereby AMPK activation (by CAMKK2) increases vascular permeability through both eNOS and p38/HSP27 phosphorylation. The study reports some interesting findings that have potentially important implications for neurovascular permeability in the retina. However, some of the reagents used are not very selective and this weakens the interpretation of the results. Additionally, there is a lack of important experimental detail for some of the studies and this needs to be resolved. Finally, some of the conclusions the authors make are difficult to rationalise and further discussion might help clarify these points.

© 2021. Published by The Company of Biologists under the terms of the Creative Commons Attribution License (https://creativecommons.org/licenses/by/4.0/).

Comments for the author

Specific Comments
1. There are no details of which isoform of AMPK alpha is being targeted for the siRNA studies. It would help interpretation of the data if the protein expression of the alpha and beta isoforms was reported. There are excellent commercial antibodies that allow for this distinction. Clearly, it is important to know which alpha isoform is being targeted (by siRNA and the beta isoforms are important for understanding the response to small molecule AMPK activators (e.g. A769662).
2. Some of the pharmacological reagents used in the study are not selective (e.g. Compound C, STO-609). Using siRNA to confirm the findings with the pharmacological reagents is extremely important in this regard (but it is important to define what the siRNA reagents are!). It is a bit disappointing that the authors use A769662. There are much more potent and selective AMPK activators available these days.
3. Is the link from AMPK to increased p38 phosphorylation known?
4. Is the pathway from CAMKK2/AMPK to VE-cad Y685 phosphorylation known (which appears to involve eNOS)?
5. The authors appear to rule out the potential for a role of PAK/cdc42 in the pathway. Is there any experimental evidence that PAK phosphorylation is not affected in this system? AMPK has been reported previously to phosphorylate PAK isoforms, so this seems a possibility? Non-selective and selective PAK isoform inhibitors are available. Do these have any impact on permeability in this system?
6. It is not clear to me how the non-redundant effects of eNOS and the p38/HSP27 pathways operate. Do the authors have any thoughts that might explain this?

First revision

Author response to reviewers' comments

Reviewer 1 Advance summary and potential significance to field...

Vascular permeability is of high clinical significance. Early signs for diabetic or hypertensive retinopathy are areas with increased vascular permeability. The secondary endpoint of the anti-VEGF-A therapy for wet age-related macular degeneration, macular thickness, is based on the assumption of VEGF-A driven vascular permeability. However, although the concept of vascular permeability is proven in the clinic, its physiological base is not understood. The manuscript by Dragoni and colleagues presents work that identifies a central VEGF-A- and bradykinin-driven pathway that results in increased permeability in the endothelial cell-to-cell junctions. Using direct measurements of vascular permeability the paper presents interesting data from well performed experiments that fully support the final conclusions.

We would like to thank this reviewer for their time and comments to improve our manuscript.

Reviewer 1 Comments for the author...

Minor comments:
1. The permeability increase by VEGF-A or BK: The authors present a couple of data of permeability increase by VEGF-A from different rows of experiments that focus on the different levels of signal transduction. In almost all panels the mean permeability increase was in between 0.55 and 0.6 that fits nicely into the variance. However, in figures 6C and 6D VEGF-A leads to permeability increases by 1.6 and 0.3 respectively. Are these differences caused by the experimental conditions that did not become clear from the figure caption? Or is there a mistake in the y-axis labeling? The same applies for the effects of BK that were between 0.3 and 0.5. However in the figures 6H and 6F the BK effects were at 0.8 and 1.2 respectively. May be in general that figure legends could be improved for readability.

© 2021. Published by The Company of Biologists under the terms of the Creative Commons Attribution License (https://creativecommons.org/licenses/by/4.0/).

The reviewer is absolutely correct and we are very grateful for notifying us of this problem: Whilst there is some variation in baseline permeability between preparations and vessels, the permeability data in these panels was not within the range of all other measurement. We are unsure what caused this erroneous data set and have therefore repeated these experiments entirely. The new data were within the variance set by all other experiments and is shown in the new panels 6 C, D, H and K.

2. Statistics: The statistics chapter in the Methods section state that there were used four independent experiments but the figure legends state that these were three independent experiments. Furthermore, the dots in the box plot indicate here and there higher “n-numbers” than three. Can it be that from each of the three independent experiments not only one but several blood vessels were analyzed?

We apologise for some inaccurate descriptions:
In the ex vivo retina, each permeability data point represents an independent biological replicate from a separate retina. Each experiment has been performed as at least 3 independent biological replicates. However, most have been repeated considerably more often.
All western blot experiments have been performed three times and each independent experiment is represented by one data point.
By contrast, the data points in Figure 1G are measurements from across three independent experiments.
Clarifying statements have now been added to the statistics paragraph in the methods section and the figure legends.

3. Westernblots: As the authors show only very small parts of a complete westernblot it would be useful to add complete blots (from each AMPKalpha, tubulin, GAPDH one blot) to the supplemental material to demonstrate that the antibodies work well and specifically (only one band at the right molecular mass marker).
We agree that data transparency is an important element of today's publication process. Representative raw scans of all immunoblot experiments are now included as supplemental material.

Reviewer 2 Advance summary and potential significance to field...

The manuscript by Dragoni et al uses an ex vivo model of retina explants to investigate the role of AMPK in neurovascular permeability. The authors confirm previous studies showing that VEGF and bradykinin activate AMPK via CAMKK2 signalling, and report that this leads to increased vascular leakage. Downstream of AMPK, increased phosphorylation of eNOS, p38, HSP27 and VE-cadherin was observed. Using mainly pharmacological approaches, the authors show that inhibition of CAMKK2, eNOS or p38 reduces VEGF and bradykinin induced permeability. Based on their findings, the authors suggest a model whereby AMPK activation (by CAMKK2) increases vascular permeability through both eNOS and p38/HSP27 phosphorylation. The study reports some interesting findings that have potentially important implications for neurovascular permeability in the retina. However, some of the reagents used are not very selective and this weakens the interpretation of the results. Additionally, there is a lack of important experimental detail for some of the studies and this needs to be resolved. Finally, some of the conclusions the authors make are difficult to rationalise and further discussion might help clarify these points.

We are very grateful for the time and critical thoughts this reviewer has provided. We are confident that, with the additional experiments, changes to the text, and explanations detailed below, the robustness of our data and their conclusion has been sufficiently improved, and thus all of the comments adequately addressed.

Reviewer 2 Comments for the author...

Specific Comments
1. There are no details of which isoform of AMPK alpha is being targeted for the siRNA studies. It would help interpretation of the data if the protein expression of the alpha and beta isoforms was reported. There are excellent commercial antibodies that allow for this distinction. Clearly, it is

© 2021. Published by The Company of Biologists under the terms of the Creative Commons Attribution License (https://creativecommons.org/licenses/by/4.0/).

important to know which alpha isoform is being targeted (by siRNA and the beta isoforms are important for understanding the response to small molecule AMPK activators (e.g. A769662).

RNAseq data sets indicate that AMPK gene expression in brain and retinal is restricted to the alpha1 isoform (with no or very little alpha2). These data sets are now cited (lines 221-222). They constituted the rationale for targeting alpha1 with our siRNA approach. We have now, as requested, also added data using alpha1-specific antibodies to show that this approach is specific (Figure 4G-I). Furthermore, our previous experiments using pan-alpha antibodies (now shown in Supplemental Figure 1H, I) show that alpha1 siRNA targets the predominant form of AMPK in the retina. We have also amended the discussion to clarify that our results are specific for alpha1 (line 348).
With regards to relevance of the AMPK activators used, retinal endothelial cells express beta1 and beta2 regulatory subunits (as now cited in line 231). We have added additional activator experiments as detailed in the next section.

2. Some of the pharmacological reagents used in the study are not selective (e.g. Compound C, STO-609). Using siRNA to confirm the findings with the pharmacological reagents is extremely important in this regard (but it is important to define what the siRNA reagents are!). It is a bit disappointing that the authors use A769662. There are much more potent and selective AMPK *activators available these days.*

We entirely agree that many pharmacological reagents are not selective. Therefore, it was vital for our study to show specificity for AMPK and back up all key experiments using CC by using highly specific siRNA. In fact, we noted that such siRNA data was missing from Figure 6B and these data have now been added as Figure 6C.
Our data shows unambiguously that Ca2+ is at the origin of both VEGF and BK-induced permeability, and that this crucially activates AMPK. To date the only known link between Ca2+ and AMPK is CAMKK, for which we provide some (arguably not completely conclusive) evidence. However, it should be noted that AMPK and its downstream targets, not CAMKK are the focus of our study. Therefore, mention of CAMKK was also removed from the shortened abstract.
The reviewer is correct that more activators of AMPK exist. They can broadly be grouped into (i) indirect activators of AMPK, (ii) AMP analogues, and (iii) compounds that bind to a unique ADaM site. Compounds of group i are considered unspecific and context-dependent and should not be used to study AMPK directly. AMP analogues (group ii) bind to the alpha subunit. Despite also affecting enzymes other than AMPK, AICAR is the paradigm compound within this group. The only more specific agonist in this group is the recently developed C13. Unfortunately, it is currently not yet available to the wider scientific community. We were close to getting it from Grahame Hardie (Dundee University) but Covid has put a temporary stop to their synthesis capacity. A769662 is one of many group iii compounds. Compared to other compounds in this group, its only disadvantage appears to be that is somewhat less potent; presumably because it is beta1 subunit specific, and as such may not work on all cellular AMPK complexes. It demonstrably does not work in cells that do not express beta1. We previously used A769662, since we inferred from brain endothelial cell expression data that retinal endothelial cells only express the beta1 subunit. However, very recent RNAseq from retinal endothelial cells shows that they express both, beta1 and beta 2 (Lipski et al., 2020). Thus, we have performed additional experiments using beta1/2-specific ADaM binders, namely MK8722 and PF739. They both stimulated retinal permeability by factors of 5 and 6.5-fold, respectively (i.e. more strongly than A769662), as well as downstream signalling including activation of p38 and eNOS (new Figure 5). The previous data using AICAR and A769662 is now shown in Supplemental Figure 2.
We are grateful that the reviewer has raised this, thus allowing us to address this obvious weakness of our study.

3. Is the link from AMPK to increased p38 phosphorylation known?

In short, no. The only two molecules described to date potentially linking the two, are PAK and TAB1. As discussed also under 3., we have performed additional experiments using FRAX597, a specific group I PAK inhibitor, to unveil any potential role of PAKs. However, FRAX597 did not have any effect on AMPK-induced phosphorylation of p38. We therefore propose a more likely pathway involving TAB1 (lines 392-404). Extensive experimentation using isoform specific siRNA will be required to identify the exact molecular link(s) under defined functional scenarios.

© 2021. Published by The Company of Biologists under the terms of the Creative Commons Attribution License (https://creativecommons.org/licenses/by/4.0/).

4. Is the pathway from CAMKK2/AMPK to VE-cad Y685 phosphorylation known (which appears to involve eNOS)?

In response to VEGF, nitric oxide from eNOS leads to direct S-nitrosylation of beta-catenin and its release from VE-cadherin (Thibeault et al., 2010). This may allow phosphorylation (and internalisation) of VE-cadherin by Y685 kinases. Since cGMP production is also an important effect of nitric oxide production, we analysed the effect of ODQ, which inhibits the eNOS-responsive soluble guanylyl cyclase sGC. In line with our previous experiments linking eNOS to VE-cadherin via sGC during ICAM-1 signalling (Martinelli et al., 2009), we found that ODQ completely inhibited VE-cadherin phosphorylation (Supplemental Figure 3A). Thus VE-cadherin phosphorylation is likely to be affected via multiple pathways downstream of eNOS, as also discussed in the manuscript (lines 373-380).

5. The authors appear to rule out the potential for a role of PAK/cdc42 in the pathway. Is there any experimental evidence that PAK phosphorylation is not affected in this system? AMPK has been reported previously to phosphorylate PAK isoforms, so this seems a possibility? Non-selective and selective PAK isoform inhibitors are available. Do these have any impact on permeability in this system?

As discussed under 3., we have performed additional experiments using FRAX597 (shown in Supplemental Figure 3). This highly specific group I PAK inhibitor had no effect on AMPK-induced permeability and signalling, indicating that PAK is not required for acute permeability in the retina. We are aware that this is not in agreement with other reports implicating PAK/cdc42 in VEGF-induced permeability. However, it should be stated that very few of these reports address permeability in intact vessels and none in intact neural vessels. Nevertheless, we agree that more in depth studies are required to understand the exact role of p21-activated kinases in vessel leakage. This is now also discussed in the manuscript (lines 392-398).

6. It is not clear to me how the non-redundant effects of eNOS and the p38/HSP27 pathways operate. Do the authors have any thoughts that might explain this?

Whilst molecular details are still sketchy, vascular permeability is clearly regulated by a wide variety of molecular endpoints. Amongst those evidenced so far, internalisation and adhesiveness of adherens junctions, as well as the cortical contractility of the cytoskeleton are undoubtedly key processes. Many studies have shown, that these processes are not redundant and need to happen in harmony for leakage to occur, and this a commonly accepted view (e.g. Claesson-Welsh et al., 2020). Our data show that, during the induction of acute permeability, AMPK regulates; on the one hand phosphorylation (and internalisation) of VE-cadherin; on the other hand phosphorylation of HSP27 (and with it cytoskeletal actin arrangement necessary to widen the weakened adherens junctions). Clearly these are two processes that need to occur in parallel to result in an effective opening of the paracellular pathway and as such best controlled by at least one common regulator such as AMPK. We believe that our discussion reflects this view accurately.

Second decision letter

MS ID#: JOCES/2020/253179

MS TITLE: AMP-activated protein kinase is a key regulator of acute neurovascular permeability

AUTHORS: Silvia Dragoni, Bruna Caridi, Eleni Karatsai, Thomas Burgoyne, Mosharraf H Sarker, and Patric Turowski
ARTICLE TYPE: Research Article

I am happy to tell you that your manuscript has been accepted for publication in Journal of Cell Science, pending standard ethics checks.

© 2021. Published by The Company of Biologists under the terms of the Creative Commons Attribution License (https://creativecommons.org/licenses/by/4.0/).

Reviewer 1

Advance summary and potential significance to field

Regulation of vascular permeability is or outmost importance in many retinal diseases and a target for clinical Intervention. Thu, the authors have submitted a paper with high translational relevance.

Comments for the author

All comments and criticisms that have been made by the refeviewers have been sarifactorily addressed.

Reviewer 2

Advance summary and potential significance to field

The manuscript by Dragoni et al uses an ex vivo model of retina explants to investigate the role of AMPK in neurovascular permeability. The authors confirm previous studies showing that VEGF and bradykinin activate AMPK via CAMKK2 signalling, and report that this leads to increased vascular leakage. Downstream of AMPK, increased phosphorylation of eNOS, p38, HSP27 and VE-cadherin was observed. Using mainly pharmacological approaches, the authors show that inhibition of CAMKK2, eNOS or p38 reduces VEGF and bradykinin induced permeability. Based on their findings, the authors suggest a model whereby AMPK activation (by CAMKK2) increases vascular permeability through both eNOS and p38/HSP27 phosphorylation. The study reports some interesting findings that have potentially important implications for neurovascular permeability in the retina.

Comments for the author

The authors have done a good job revising their manuscript and have addressed all of my concerns. The inclusion of more potent and selective AMPK activators is particularly welcome.

A couple of very minor points:
1. I presume the studies the authors cite for AMPK subunit expression (e.g. line 223 and 233) are based on mRNA profiling? Clarifying this point would be good.

2. The wording for the AMPK activators (line 236) is slightly confusing. The Pfizer and Merck activators are "pan-beta" or non-selective (the way it is currently written it could be mistaken for assuming PF739 is b1 and MK8722 is b2 specific. Also, A769662 is not b1 specific, just more potent against b1. There is a nice recent review covering the pharmacological activation of AMPK that the authors might like to cite in addition to the Hardie 2017 ref (on line 235): Steinberg and Carling (2019), Nat. Rev. Drug Disc. 18, 527-551.

© 2021. Published by The Company of Biologists under the terms of the Creative Commons Attribution License (https://creativecommons.org/licenses/by/4.0/).
